# Association Between Geographic Location and Radiotherapy Treatment Delay in Head and Neck Squamous Cell Carcinoma: A Retrospective Study

**DOI:** 10.7759/cureus.73253

**Published:** 2024-11-07

**Authors:** Kohei Matsumoto, Yukiko Honda, Takahiro Maeda, Yoshihiko Kumai

**Affiliations:** 1 Department of Otolaryngology-Head and Neck Surgery, Nagasaki University Graduate School of Biomedical Sciences, Nagasaki, JPN; 2 Department of Otolaryngology-Head and Neck Surgery, Nagasaki Goto Chuoh Hospital, Nagasaki, JPN; 3 Department of Social Epidemiology, Graduate School of Medicine and School of Public Health, Kyoto University, Kyoto, JPN; 4 Department of General Medicine, Nagasaki University Graduate School of Biomedical Sciences, Nagasaki, JPN

**Keywords:** chemoradiotherapy, geographic location, head and neck squamous cell carcinoma, radiotherapy waiting period, treatment delays

## Abstract

In head and neck squamous cell carcinoma (HNSCC) treatment, extended waiting times for chemoradiotherapy negatively impact survival outcomes. Identifying contributing factors to these delays and strategies to minimize them could improve the prognosis for HNSCC patients. However, the factors affecting treatment delays remain incompletely understood, warranting further investigation. This retrospective study examined the association between patient residence and treatment delay. Medical records of 125 patients with squamous cell carcinoma of the oropharynx, hypopharynx, or larynx, treated with radiotherapy or chemoradiotherapy at the Department of Otolaryngology, Nagasaki University School of Medicine, were analyzed. Treatment delay was defined as the period from the patient’s initial medical visit to the start of treatment at our hospital. The relationship between factors, such as patient location and waiting period, was assessed. Uni- and multivariate logistic regression analyses revealed that residing in a remote area was significantly associated with longer treatment delays (OR: 4.41, 95% CI: 1.40-13.91, p = 0.01; adjusted OR: 7.60, 95% CI: 1.93-29.98, p < 0.01). These findings underscore the need for interventions to reduce treatment delays in HNSCC patients from remote areas.

## Introduction

In head and neck squamous cell carcinoma (HNSCC), increasing tumor volume correlates with lower tumor control rates [1]. A study comparing diagnostic CT images with those used for radiotherapy planning in the same patients found a tumor volume increase in 70% of cases when more than 28 days elapsed between imaging sessions [2]. Numerous studies on chemoradiotherapy for HNSCC have also demonstrated that extended waiting periods for treatment adversely affect prognosis [3-6]. Identifying factors contributing to treatment delays and exploring ways to reduce these waiting times could improve outcomes in HNSCC. Previous studies have categorized delays as patient-related or physician-related [7]. Patient-related factors include fear, hesitation toward medical care, and limited awareness of the disease [8], while physician-related factors include misdiagnosis and insufficient knowledge [9,10]. Other factors associated with prolonged waiting periods include cancers in anatomically challenging sites [11,12] and initial consultations with general practitioners (GPs) [13]. However, research specifically on treatment delays in HNSCC remains limited. Given the unique geographic context of our study area, which includes several remote islands, we aimed to examine the relationship between patient residence and treatment delay - an aspect not previously explored in depth. We also investigated the association between the department of first medical contact and treatment delays within our hospital’s catchment area, expanding on insights from earlier studies.

## Materials and methods

Study sample

This retrospective study analyzed medical records from the Department of Otolaryngology, Nagasaki University School of Medicine. The study population included patients diagnosed with squamous cell carcinoma of the oropharynx, hypopharynx, or larynx between April 1, 2013, and March 31, 2019, who were eligible for radiotherapy or chemoradiotherapy. Excluded were patients treated at the Department of Radiology or other institutions, as well as cases of cancer detected during follow-up after previous head and neck cancer treatment, cases of recurrence diagnosed in follow-up, and patients with overlapping cancers prioritizing treatment for other malignancies. Additionally, patients who prioritized treatment of comorbid conditions, such as myocardial infarction, were excluded to focus on those who could pursue timely HNSCC treatment. This study received approval from the Research Ethics Committee of Nagasaki University Hospital (approval number 20051818).

The general diagnostic and treatment initiation process within our hospital’s medical network aligns with standard practices across Japan. Initially, patients consult private clinics of their choice. Those suspected of HNSCC are referred to our hospital for further assessment. Here, cancer staging is established via imaging, and diagnosis is confirmed through pathology. After all diagnostic results are gathered, a multidisciplinary conference determines the treatment approach, which is then implemented. Patients residing further from our hospital typically undergo initial imaging and histopathological testing at their nearest base hospital.

Outcome

The primary outcome was treatment delay, defined as the period from the starting point (the date of the patient’s initial visit to the referring medical institution) to the start of radiotherapy at the Otolaryngology Department of Nagasaki University Hospital. Initially recorded as a continuous variable, treatment delay was later categorized into two groups based on the median value: patients with a short delay (≤35 days) and those with a long delay (≥36 days). This classification was based on evidence that tumor volume increases in 70% of cases if waiting time exceeds 28 days from diagnosis [2]. Additionally, studies consistently indicate that delayed treatment initiation correlates with a poorer prognosis, although the degree of impact varies across studies [3-6]. Previous research on the time from pathological diagnosis to treatment initiation reports median delays of 35 [14] and 37 [6] days, making the designation of ≥36 days as a long delay both clinically and empirically justified.

Exposure variable

Residence

**Figure 1:**
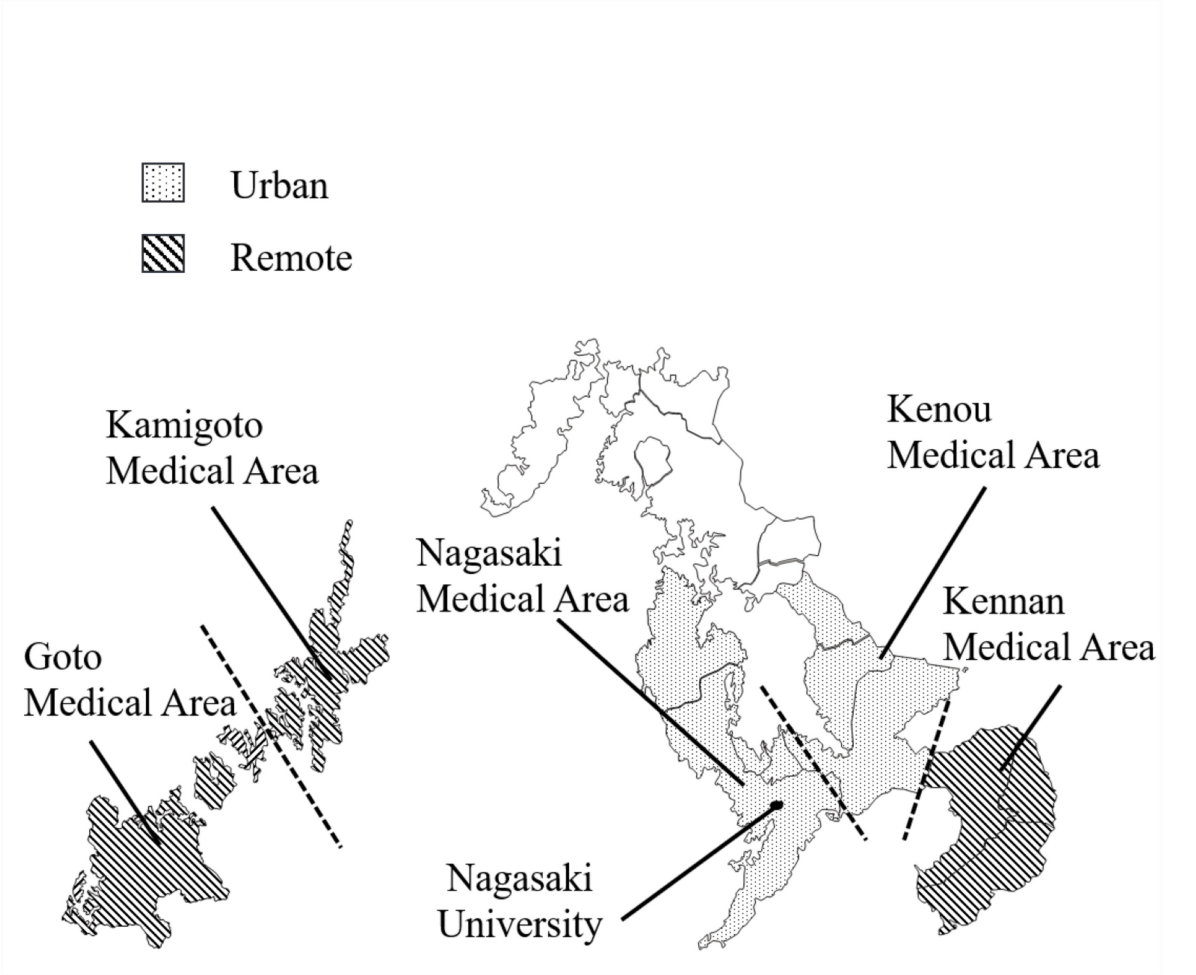
Map of Nagasaki prefecture highlighting the medical service areas associated with our hospital The distance and travel time from Nagasaki University Hospital to the base hospitals in each medical district are as follows: Goto Medical Area: 100 km, two to three hours one way by ship; Kamigoto Medical Area: 100 km, two to three hours one way by ship; Kenou Medical Area: 25 km, 35 minutes one way by car; and Kennan Medical Area: 65 km, two hours 20 minutes one way by train.

Figure 1 displays a map of our hospital’s medical service area, with patient residences categorized into urban and remote based on the medical area associated with their address. The Department of Otolaryngology at Nagasaki University School of Medicine covers five medical regions: Nagasaki, Kenou, Goto, Kamigoto, and Kennan.

Our hospital, located in the Nagasaki Medical Area, benefits from ample medical resources and convenient access. The adjacent Kenou Medical Area also has good accessibility to our hospital and is well-resourced, including a full-time ENT specialist at its base hospital. In contrast, the Goto and Kamigoto Medical Areas are remote islands approximately 100 km from our hospital, with limited medical resources and no full-time ENT specialists. The Kennan Medical Area, situated on the mountainous Shimabara Peninsula, is connected to the Nagasaki Medical Area via the Kenou region, yet its base hospital is about 70 km from ours. This distance, combined with insufficient medical resources and no full-time ENT specialist, limits access. Given these factors, patient residences were classified as urban or remote for analysis.

Confounders

Confounders in this study included several factors such as demographic variables (age and sex), medical characteristics (primary site, stage, and irradiation method), and social factors (department of initial visit, duration of treatment delays, and receipt of welfare benefits).

Age

Age was categorized into two groups based on the median: 69 years or younger, and 70 years or older.

Primary Site

Primary site was classified into three groups: oropharynx, hypopharynx, and larynx. This classification is based on studies indicating that head and neck cancers in anatomically challenging locations often experience longer treatment delays [11,12].

Stage

Stage was categorized into two groups: Stage I/II and Stage III/IV. Stage I/II typically represents early-stage cancer, while Stage III/IV indicates advanced cancer. This classification is commonly used in clinical practice and in some previous studies [10,13].

Irradiation Method

Irradiation method was categorized into two groups: intensity-modulated radiation therapy (IMRT) and non-IMRT. IMRT typically requires more time for planning the irradiation field compared to non-IMRT.

For social factors, adjustments were made based on the department of the first visit, the presence of any prolonged treatment delays, and whether the patient received welfare benefits.

Department of First Visit

Department of first visit was categorized into two groups: ENT and non-ENT. This classification is based on findings that visiting a GP can lead to longer treatment delays [13].

Long-Term Closure Period of Hospital

Patients were divided into two groups based on whether their clinic experienced a long-term closure during the waiting period. Specifically, patients were classified as having experienced long-term closures if the hospital was closed for four or more consecutive days during the treatment delay. This classification aimed to assess the impact of hospital closures on treatment delays. While there are no prior studies defining a long-term closure period, mental health literature often considers holidays of four or more days as significant [15]. At our hospital, closures of four or more consecutive days typically occur during the May holidays and the New Year period. During the study period, the May holidays lasted about four days, and the New Year holidays lasted about seven days. Therefore, defining a long-term closure as any period with four or more consecutive days of closure during the treatment delay was deemed appropriate.

Patients Receiving Welfare Benefits

Patients were categorized into two groups based on whether or not they received welfare benefits. Previous studies have examined income level as a factor influencing treatment delay [6,9]. We used welfare status as an indicator of the patient’s income level. In Japan, public assistance is a system that provides financial support to individuals in poverty, with eligibility based on an annual household income below a government-defined threshold. Therefore, using welfare status as a criterion for low-income status was considered appropriate.

Statistical analysis

Patients were first categorized into two groups based on the length of their treatment delay, and the distribution of each variable within these groups was analyzed descriptively. Differences in distribution between the groups were assessed using the Chi-square test for the primary site and Fisher’s exact test for other variables. The relationship between treatment delay (outcome) and residence (exposure variable) was analyzed using bivariate logistic regression. This analysis also examined the relationship between the outcome and other covariates. Additionally, multivariate logistic regression was employed to assess the association between the outcome and the exposure variable while adjusting for confounding factors. For sensitivity analysis, Model 1 was created, adjusting for biological factors only (age, sex, primary site, and stage). Model 2 further included environmental factors (hospital closure and welfare assistance) and adjusted for them. Multivariate logistic regression was performed for each model to evaluate the link between longer waiting periods and residence. A significance level of p < 0.05 was used for all statistical tests. Statistical analysis was conducted using JMP Pro 14.2.0 (JMP Statistical Discovery LLC, Cary, NC, USA).

## Results

**Figure 2:**
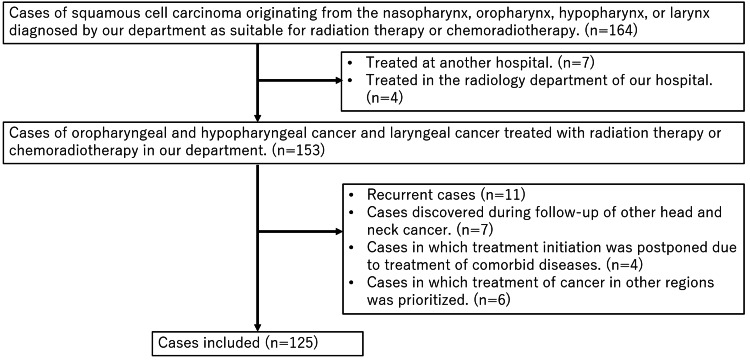
Flowchart of case selection for the study Initially, 164 cases of squamous cell carcinoma from the nasopharynx, oropharynx, hypopharynx, or larynx were eligible for radiotherapy or chemoradiotherapy. Four nasopharyngeal cases were excluded as they were treated by the radiology department. Seven cases treated elsewhere were also excluded. Eighteen cases in circumstances that would have led to earlier detection than usual were excluded. Ten cases in which early intervention was precluded for various reasons were also excluded. The final analysis included 125 cases of squamous cell carcinoma from the oropharynx, hypopharynx, or larynx treated at our department.

This study included 164 patients, of whom 125 were analyzed after excluding those who did not meet the criteria. The selection process for the analysis is detailed in Figure 2. The median treatment delay was 35 days, ranging from 12 to 289 days. Further details are provided in the Appendices. Patients were divided into two groups based on this median: those with a waiting period of 35 days or less were classified as the short treatment delay group, and those with a treatment delay of 36 days or more were classified as the long treatment delay group.

Table 1 shows the characteristics of patients categorized by treatment delay. Patients with longer treatment delays were more likely to be from remote areas (24.2% vs. 7.9%, p = 0.02), to have initially visited a non-ENT department (56.5% vs. 19.0%, p < 0.01), to have hypopharyngeal cancer (43.5% vs. 17.5%, p < 0.01), and to have experienced longer hospital closures (27.4% vs. 6.3%, p < 0.01).

**Table 1:** Characteristics of patients categorized by treatment delay ^a^ p-values from Fisher’s exact test ^b^ p-values from the Chi-square test Bold signifies p < 0.05. IMRT: intensity-modulated radiation therapy

Variable		Short treatment delay (≤35 days, n = 63)	Long treatment delay (≤36 days, n = 62)	p-value
		n (%)	n (%)	
Age	<70	28 (44.4)	34 (54.8)	0.29^a^
	≥70	35 (55.6)	28 (45.2)	
Sex	Male	60 (95.2)	59 (95.2)	1:00 am
	Female	3 (4.8)	3 (4.8)	
Primary site	Larynx	38 (60.3)	20 (32.3)	<0.01^b^
	Oropharynx	14 (22.2)	15 (24.2)	
	Hypopharynx	11 (17.5)	27 (43.5)	
Department of first visit	ENT	51 (81.0)	27 (43.5)	<0.01^a^
	Non-ENT	12 (19.0)	35 (56.5)	
Stage	1.2	32 (50.8)	24 (38.7)	0.21^a^
	3.4	31 (49.2)	38 (61.3)	
Patients receiving welfare benefits	Received	5 (7.9)	6 (9.7)	0.76^a^
	Non-received	58 (92.1)	56 (90.3)	
Residence	Urban	58 (92.1)	47 (75.8)	0.02^a^
	Remote	5 (7.9)	15 (24.2)	
Long-term closure period of hospital	Yes	4 (6.3)	17 (27.4)	<0.01^a^
	No	59 (93.7)	45 (72.6)	
Irradiation method	IMRT	18 (28.6)	26 (41.9)	0.14^a^
	Non-IMRT	45 (71.4)	36 (58.1)	

Table 2 presents the results of both bivariate and multivariate analyses investigating the relationship between treatment delay and various factors. In the bivariate analysis, a longer treatment delay was significantly associated with residing in a remote area (OR 3.70, 95% CI 1.25-10.93, p = 0.02). This association remained significant after adjusting for age, primary site, department of first consultation, stage, welfare assistance status, long hospital closures during the treatment delay, and irradiation method (AOR 7.60, 95% CI 1.93-29.98, p < 0.01). Moreover, a longer treatment delay was significantly linked to initially visiting a non-ENT department (OR 5.51, 95% CI 2.52-12.7, p < 0.01). This relationship persisted after adjusting for age, primary site, stage, welfare benefits, residence, long hospital closures during the treatment delay, and irradiation method (AOR 7.27, 95% CI 2.60-20.36, p < 0.01).

**Table 2:** Bivariate and multivariate analysis of factors influencing treatment delay Bold signifies p < 0.05. IMRT: intensity-modulated radiation therapy

Variable		OR	95% CI	p-value	AOR	95% CI	p-value
Age	<70	Reference			Reference		
	≥70	0.66	(0.33-1.33)	0.25	0.42	(0.16-1.12)	0.08
Sex	Male	Reference			Reference		
	Female	1.02	(0.20-5.24)	0.98	0.38	(0.05-2.91)	0.35
Primary site	Larynx	Reference			Reference		
	Oropharynx	2.04	(0.82-5.05)	0.12	0.67	(0.11-4.15)	0.67
	Hypopharynx	4.66	(1.92-11.31)	<0.01	3.29	(0.76-14.20)	0.11
Department of first visit	ENT	Reference			Reference		
	Non-ENT	5.51	(2.46-12.32)	<0.01	7.27	(2.60-20.36)	<0.01
Stage	1.2	Reference			Reference		
	3.4	1.63	(0.81-3.35)	0.17	0.55	(0.14-2.21)	0.4
Patients receiving welfare benefits	Received	Reference			Reference		
	Non-received	0.8	(0.23-2.79)	0.73	1.02	(0.22-4.59)	0.98
Residence	Urban	Reference			Reference		
	Remote	3.7	(1.25-10.93)	0.02	7.6	(1.93-29.98)	<0.01
Long-term closure period of hospital	No	Reference			Reference		
	Yes	5.57	(1.75-17.71)	<0.01	9.58	(2.42-37.97)	0.03
Irradiation method	IMRT	Reference			Reference		
	Non-IMRT	0.55	(0.26-1.17)	0.12	0.24	(0.06-0.97)	0.04

Multivariable logistic regression analysis demonstrated that both residence and the department of first consultation were significantly associated with longer treatment delays. Further analysis of these two factors was conducted using Model 1 and Model 2, as outlined earlier. The adjusted ORs are presented in Table 3. In both models, residence and department of first consultation remained significantly associated with extended treatment delays.

**Table 3:** Adjusted ORs and 95% CI for factors influencing treatment delay Model 1: adjusted for age, sex, primary tumor department, and stage; Model 2: Model 1 + receipt of public assistance, difficulty seeing a doctor due to long-term closure Bold signifies p < 0.05. IMRT: intensity-modulated radiotherapy

Variable		Model 1			Model 2		
		AOR	95% CI	p-value	AOR	95% CI	p-value
Age	<70	Reference			Reference		
	≥70	0.65	(0.30-1.41)	0.28	0.56	(0.24-1.29)	0.18
Sex	Male	Reference			Reference		
	Female	0.71	(0.12-4.13)	0.7	0.92	(0.16-5.26)	0.92
Primary site	Larynx	Reference			Reference		
	Oropharynx	3.45	(0.91-13.06)	0.07	3.33	(0.85-13.00)	0.08
	Hypopharynx	8.08	(2.27-28.71)	<0.01	7.28	(1.99-26.67)	<0.01
Department of first visit	ENT	Reference			Reference		
	Non-ENT	4.5	(1.90-10.66)	<0.01	5.03	(2.04-12.40)	<0.01
Stage	1.2	Reference			Reference		
	3.4	0.46	(0.14-1.53)	0.2	0.58	(0.17-2.00)	0.39
Patients receiving welfare benefits	Received	Reference			Reference		
	Non-received	1.04	(0.26-4.20)	0.95	1.12	(0.27-4.73)	0.87
Residence	Urban	Reference			Reference		
	Remote	4.41	(1.40-13.91)	0.01	5.25	(1.55-17.86)	<0.01
Long-term closure period of hospital	No	Reference			Reference		
	Yes	6.6	(1.88-23.17)	<0.01	6.62	(1.88-23.25)	<0.01
Irradiation method	IMRT	Reference			Reference		
	Non-IMRT	0.61	(0.19-1.95)	0.4	0.54	(0.16-1.80)	0.32

## Discussion

In this study, residing in a remote area was significantly associated with a longer treatment delay. Previous research in other medical fields has indicated that the distance to a treatment facility is a factor contributing to delayed treatment initiation [16]. We believe this also applies to HNSCC, where the distance to a treatment facility plays a role in delaying care. The remote areas included in this study cover several distant islands, where travel to Nagasaki University Hospital typically requires a two- to three-hour boat ride, which can be burdensome for patients. Additionally, cancellations due to weather conditions further exacerbate the challenge. Clinically, patients from remote islands often express a sense of treatment delays, making the study findings reasonable. Addressing the travel burden on patients in remote areas is a key challenge. The introduction of telemedicine has been reported to alleviate this burden [17,18], and studies show that patients are highly satisfied with telemedicine services [19]. For facilities like ours, serving a large rural area, telemedicine could be considered to reduce travel burdens and potentially shorten treatment delays.

This study also found that visiting a non-ENT department for the first consultation was significantly linked to longer treatment delays. Previous studies have shown that delays can occur when GPs are involved in the initial cancer diagnosis. Even if GPs do not immediately recognize HNSCC, early diagnosis can still occur through follow-up visits. However, if GPs do not identify HNSCC and do not arrange follow-up visits, treatment delays may result [13,20]. Since GPs frequently encounter patients with symptoms similar to HNSCC but rarely diagnose actual cases, this may contribute to delays [21,22]. Additionally, initial doctors may misinterpret symptoms such as cervical lymphadenopathy as benign or indicative of infection [10]. Improving coordination between hospitals and GPs in Japan could help facilitate quicker referrals to otolaryngology for suspected HNSCC. Further research on the characteristics of HNSCC patients who first visit non-ENT departments could help identify specific symptoms and patient characteristics that warrant closer attention.

In this study, the primary site was significantly associated with treatment delay. Laryngeal cancer often presents with early, clear symptoms, such as hoarseness, making early diagnosis and treatment easier. In contrast, hypopharyngeal cancer can present with non-specific symptoms, such as a sore throat or difficulty swallowing, which may not suggest cancer. As these symptoms are not unique to cancer, identifying hypopharyngeal lesions early can be challenging, potentially contributing to treatment delays.

The study also found that including four or more consecutive days of hospital closure during the treatment waiting period was significantly associated with treatment delay. This includes holidays such as the New Year and the long May holidays, which last between four and seven days. Although delays beyond these periods should not occur, the median treatment delay increased by 25 days when a long closure period was factored in. This suggests that while the closure periods themselves do not directly extend the waiting period, they contribute to an overall increase in the treatment delay by extending the total waiting time. The correlation between long closure periods and treatment delays is notable, but its overall impact is limited.

Limitations

Definitions of treatment delay vary across studies. Some studies define treatment delay as beginning from the date the patient first notices symptoms. In our retrospective study, it was difficult to pinpoint the exact date when patients became aware of their symptoms based on the available medical records. As a result, we defined the treatment delay as starting from the date the patient first visited any medical institution. This approach may lead to an incomplete analysis of patient-related factors, as some factors found to be unrelated to treatment delay in our study could be significantly associated if we had considered the patient’s awareness of symptoms. Another limitation of our study is the comparison of our findings with those from studies that use different definitions of treatment delay.

## Conclusions

In this study, residing in a remote area was significantly associated with longer treatment delays. Additionally, starting treatment at a non-ENT department was also linked to extended delays. Future research should explore the impact of residence location on patient prognosis. Furthermore, efforts should focus on reducing treatment delays, particularly for patients living in remote areas, to improve outcomes.

## Appendices

**Table 4:** Median treatment delay by patient characteristics * According to Wilcoxon's rank sum test

	N (%)	Median treatment delay	p*
Age			0.18
<70	62 (49.6)	38.5	
≥70	63 (50.4)	35	
Sex			0.47
Male	119 (95.2)	35	
Female	6 (4.8)	34.5	
Primary site			<0.01
Oropharynx	29 (23.2)	36	
Hypopharynx	38 (30.4)	52	
Larynx	58 (46.4)	33	
Department of first visit			<0.01
ENT	78 (62.4)	33	
Non-ENT	47 (37.6)	50	
Stage			0.07
1.2	56 (44.8)	35	
3.4	69 (55.2)	38.5	
Patients receiving welfare benefits			0.5
Yes	11 (8.8)	40	
No	114 (91.2)	35	
Residence			0.06
Urban	105 (84)	35	
Remote	20 (16)	51	
Long-term closure period of hospital			<0.01
Yes	21 (16.8)	60	
No	104 (83.2)	35	
Irradiation method			0.38
IMRT	44 (35.2)	39.5	
Not IMRT	81 (64.8)	35	
